# Study of Injection Molding Process to Improve Geometrical Quality of Thick-Walled Polycarbonate Optical Lenses by Reducing Sink Marks

**DOI:** 10.3390/polym16162318

**Published:** 2024-08-16

**Authors:** Jiri Vanek, Martin Ovsik, Michal Stanek, Jan Hanzlik, Vladimir Pata

**Affiliations:** Faculty of Technology, Tomas Bata University in Zlin, Vavreckova 5669, 760 01 Zlin, Czech Republic; vanek@utb.cz (J.V.); stanek@utb.cz (M.S.); j_hanzlik@utb.cz (J.H.); pata@utb.cz (V.P.)

**Keywords:** injection molding, polycarbonate, thick-walled parts, optical lenses, quality improvement, process evaluation

## Abstract

This study investigates the challenges and potential of conventional injection molding for producing thick-walled optical components. The research primarily focuses on optimizing process parameters and mold design to enhance product quality. The methods include software simulations and experimental validation using polycarbonate test samples (optical lenses). Significant parameters such as melt temperature, mold temperature, injection pressure, and packing pressure were varied to assess their impact on geometric accuracy and visual properties. The results show that lower melt temperatures and higher mold temperatures significantly reduce the occurrence of dimensional defects. Additionally, the design of the gate system was found to be crucial in minimizing defects and ensuring uniform material flow. Effective packing pressure was essential in reducing volumetric shrinkage and sink marks. Furthermore, we monitored the deviation between the predicted and actual defects relative to the thickness of the sample wall. After optimization, the occurrence of obvious defects was eliminated across all sample thicknesses (lenses), and the impact of the critical defect, the sink mark on the planar side of the lens, was minimized. These findings demonstrate the substantial potential of conventional injection molding to produce high-quality thick-walled parts when these parameters are precisely controlled. This study provides valuable insights for the efficient design and manufacturing of optical components, addressing the growing demand for high-performance thick-walled plastic products.

## 1. Introduction

Injection molding technology is an advanced method for processing plastics that is widely used across various industries. The versatility of this production process supports the manufacturing of most plastic products. While most injection-molded parts are thin-walled, there is an increasing demand for thicker components, particularly in automotive lighting, which requires high precision and optical clarity. As demand rises, applications expand into fields like sensor engineering, lighting, and projection technologies. Producing thick-walled optical parts via injection molding poses significant challenges, including achieving precise geometric accuracy, surface quality, and managing internal stresses affecting optical performance. Conventional injection molding holds untapped potential for certain parts but remains understudied, necessitating further research to explore its capabilities fully.

Researchers have investigated producing plastic parts with substantial wall thickness, exploring various techniques and materials. For instance, publications [1,2,3,4,5] discuss manufacturing thick-walled lenses and optical components using multilayer injection molding methods, achieving high shape and dimensional accuracy. This process requires specialized machines, molds, and optimized parameters. Further studies [6,7,8] involve numerical and experimental analyses of conventional injection molding for thick-walled parts, aiming to limit cavity formation. These studies employed injection molding with compression, requiring specialized molds and machines. Additional research [9,10,11,12] has focused on optical aspects rather than the broader manufacturing processes, emphasizing how parameters influence optical properties. Alternative methods combining multilayer injection molding and compression techniques were investigated in publication [13], producing a triangular optical element with minimized shrinkage and the desired optical properties. Other studies [14,15,16] examined thick-walled non-transparent parts using conventional injection molding, focusing on process conditions’ impact on quality indicators like deformation and shrinkage.

Research on conformal cooling systems for injection molds used for optical parts with significant wall thickness has been conducted extensively in studies [17,18,19,20,21]. These studies focused on designing and simulating cooling channels to homogenize temperature distribution, reduce deformation and internal stresses, and thereby enhance product quality. Innovative cooling channel designs allowed for uniform temperature control, improved dimensional stability, reduced cycle times, and better optical properties.

The influence of selected process parameters and wall thickness on shrinkage was analyzed using RSM (Response Surface Methodology) in publications [22,23]. The results indicated that increasing polymer melt temperature led to more intense shrinkage, while changes in mold temperature, injection pressure, hold time, and cooling time had limited effects. Study [24] suggests that RSM could optimize parameters, reduce defects, and improve efficiency.

Further refinement in optimizing injection molding conditions for thick-walled parts using GRA (Grey Relational Analysis) and other methods was explored in publications [25,26,27,28,29,30]. These studies identified optimal parameters to minimize deformation and residual stress, examining optical properties like refractive indices and light transmission. Studies [31,32] confirmed improvements in quality and efficiency through simulations. Study [33] used methods like the Taguchi method to reduce warpage and sink marks, successfully fabricating high-quality thick-plane lenses.

The design aspects of the mold gate system, specifically the gate and its geometry, were discussed in publications [34,35], aiming to optimize the design to reduce residual stress and enhance optical and geometrical parameters. Simulations in CAE software Moldex3D R13 supported these designs.

Study [36] proposed a technique for achieving higher-quality injected lenses by maintaining the desired surface profile through optimal conditions identified via CAE simulations and neural network predictions. Experimental data corresponded with these predictions, proving the technique’s applicability for precise optical lens production.

An approach for injecting concave plastic lenses into a mold cavity formed by silica inserts coated with graphene was demonstrated in article [37]. This approach yielded lower residual stress and more uniform light refractive index distribution compared to conventional materials, supported by CAE simulations.

Studies [38,39] used initial injection shrinkage and deformation values to adjust mold insert geometry, minimizing part deformation and ensuring efficient, precise production. This approach highlighted conditioning injected optical products for higher homogeneity by reducing residual stress.

Reviewing the current research reveals significant advancements and optimizations in injection molding technology, addressing the challenges of producing thick-walled, high-quality optical parts. However, a notable gap remains in understanding and applying conventional injection molding, which holds substantial potential to meet diverse application requirements. Therefore, this study aims to explore and maximize the potential of conventional injection molding, investigating its capabilities to produce high-quality thick-walled components efficiently.

## 2. Materials and Methods

### 2.1. Overview of the Experiment

The experimental design and its execution were tailored to meet the requirements for producing thick-walled optical parts as demanded by industrial practice. The design of the test samples and injection molds was supported by software simulations; this was essential due to the geometry of the test samples, which went against common design practices for plastic parts. Based on the obtained results, a more detailed understanding of the processes occurring during the production cycle in the mold was achieved, allowing the establishment of boundary conditions for the subsequent definition of technological parameters for the injection molding of test samples. To evaluate the specific impact of individual process conditions, their levels, and mutual combinations on the dimensional stability of the produced sample, the statistical method of Design of Experiment (DOE) was applied. The geometric characteristics of these samples were then compared with the desired CAD model geometries, utilizing 3D scanning principles and subsequently statistically processing the obtained data.

**Figure 1:**
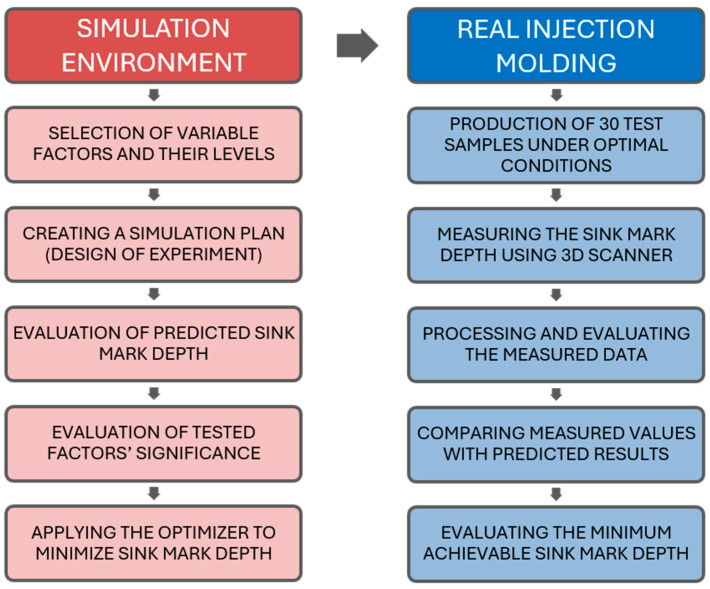
Schematic plan of the experiment.

The chosen processing methods included three-dimensional modeling, injection molding process simulations, the injection molding of test samples, the surface scanning of the manufactured samples using a laser 3D scanner, followed by data processing and evaluation, and finally the application of statistical methods and the interpretation of the results. A schematic plan of the experiment is shown in Figure 1.

### 2.2. Materials

The injection molded samples were made from polycarbonate (PC) provided by Covestro (Covestro AG, Leverkusen, Germany), commercially known as Makrolon LED 2245. A natural transparent shade without any color correction was used, which may show a slight yellow tint when viewed across an edge. This polymer is widely utilized in optical and lighting applications due to its versatility, excellent light transmission, dimensional stability, and thermal resistance. Additionally, this material exhibits good light conductivity across a broad spectrum of radiation. It also demonstrates good shape stability, temperature resistance, weather resistance, and resistance to yellowing. The tested material was dried following the parameters specified in the material datasheet, which recommended a drying temperature of 120 °C and a duration of 3 h. These conditions were specified by the material manufacturer. The granulate was then transferred into the injection molding machine via pneumatic suction.

### 2.3. Software Tools and Technical Equipment

A variety of software tools and technical equipment were utilized in this study. All design proposals, whether for test samples or injection molds, were realized using the CATIA V5 (Dassault Systemes SE, Vélizy-Villacoublay, France) program, which enabled the creation of all 3D models, assemblies, and manufacturing documentation.

In the conducted experiment, two simulation software tools specializing in injection molding analysis were used. The choice of specific software was based on the availability of licenses at the university. The use of both simulation tools, Moldflow 2023 (Autodesk, Inc., San Francisco, CA, USA) and Cadmould v17 (Simcon Kunststofftechnische Software GmbH, Würselen, Germany), allowed for the application of their unique features and the subsequent comparison of calculated data.

Test samples were prepared using an Allrounder 470 E 1000-290 Golden Electric (Arburg GmbH + Co KG, Loßburg, Germany) injection molding machine, with all parameters suitable for the purpose of the experiment. The machine was complemented with an Arburg Thermolift 100-2 dryer, to allow for the pre-injection drying of the material, and a Regloplas 150 Smart (Regloplas AG, St. Gallen, Switzerland) temperature control unit, which met all requirements regarding mold temperature control.

The surfaces of the produced samples were scanned using a Nikon MCAx30 (Nikon Corporation, Tokyo, Japan) laser 3D scanner, and the obtained data were subsequently processed and evaluated using the Polyworks 2023 (InnovMetric Software Inc., Quebec, QC, Canada) inspector tool. Finally, statistical methods were applied, and the results were interpreted using Minitab v21 (Minitab Inc., State College, PA, USA).

### 2.4. Design of Test Samples

**Figure 2:**
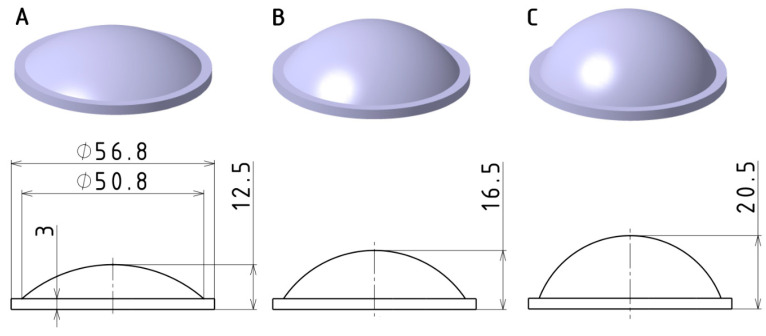
Visualization and dimensions of designed test sample variants: (**A**) 12.5 mm; (**B**) 16.5 mm; (**C**) 20.5 mm.

The test sample for the experiments was a thick-walled optical lens with a planoconvex geometry, designed in three dimensional variations (Figure 2). The lens has a positive focal length and can be used to focus collimated light, collimate light from a point source, or reduce the angle of light dispersion. Potential applications include illumination systems, optical systems for imaging or scanning, solar concentrators, and military applications. The shape and dimensions of the samples were based on the design of commercially available glass lenses, with minor modifications to facilitate production via injection molding. The geometry of the test samples was defined by a plane on one side and a convex spherical surface on the other, with the maximum thickness located at the center.

### 2.5. Experimental Injection Mold

**Figure 3:**
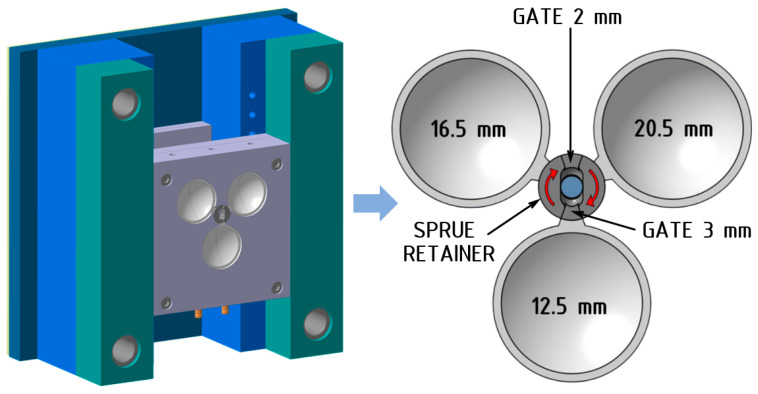
Experimental mold with a detailed view of the cavity layout relative to the sprue retainer.

The test samples were produced using an experimental injection mold with a single main parting line and a cold runner system. To facilitate the injection molding of optical lenses, a test mold was designed and manufactured with three different mold cavities and a rotating gate holder insert (Figure 3). This design allowed for the production of lenses with three different thicknesses using two gate sizes, without the need to replace the cavity-defining components.

**Figure 4:**
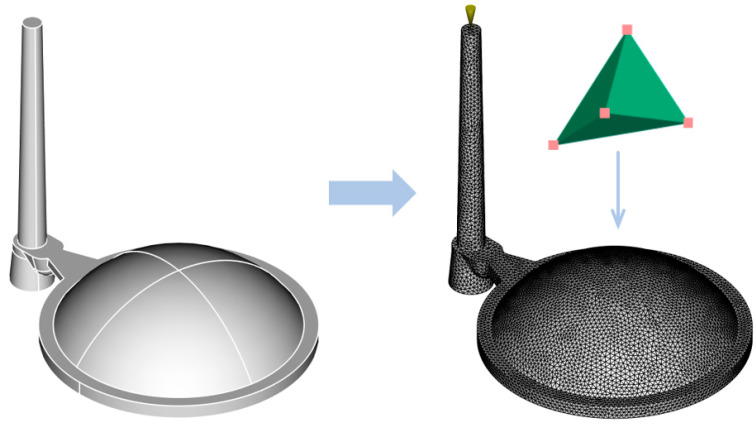
Volumetric mesh definition for imported 3D model.

The transport of the melt from the nozzle of the injection molding machine’s injection unit to the mold cavity was facilitated through a sprue bushing, which led into an adjustable sprue retainer. From there, the melt was further distributed through the selected gate into one of the mold’s cavities. Figure 4 illustrates that by rotating the sprue retainer, it is possible to define the type of gate and the mold cavity to be filled. The specific position is secured by a spring-loaded ball that engages with holes on the opposing face of the sprue retainer.

### 2.6. Preparation of Injection Molding Simulations

The simulation preparation began with importing the three-dimensional CAD model into the Moldflow 2023 software environment in STEP format, followed by the creation of a three-dimensional mesh (3D tetrahedral) representing the geometry of the injection-molded lens (Figure 4). The mesh connects the surface and volume of the product using irregular tetrahedrons with four faces, six edges, and four nodes.

Moldflow lacks built-in tools for mesh convergence analysis, so a manual study was conducted. Using default element sizes smaller than 1 mm showed minimal impact on the accuracy of the results, particularly for sink mark depth predictions, but significantly increased simulation time. Therefore, a 1 mm mesh size was chosen for optimal accuracy and computational efficiency. This size ensures the adequate coverage of geometric features without distorting results. Reducing it further would increase time and computing requirements without improving accuracy. The Advancing Layers technique was chosen as the mesh generation method because it does not rely on surface matching and offers better refinement through thickness and near brims.

The generated mesh underwent subsequent verification to ensure it met all the necessary requirements for an accurate simulation (Table 1). The primary parameter monitored was the ratio between the longest and shortest edges of the tetrahedral elements comprising the mesh. This ratio should not exceed 30, with the total maximum observed value being 16.14. The dihedral angle, which is the angle between the faces of the tetrahedra in the mesh, should ideally be lower than 178 degrees, which was safely maintained for all sample variants. Steeper angles indicate nearly flat tetrahedra, reducing the accuracy in representing the 3D part. Moreover, no overlapping elements, unconnected nodes, inverted tetras, collapsed faces, or other undesirable imperfections that could hinder the simulation or negatively impact its accuracy were identified during the verification process.

**Figure 5:**
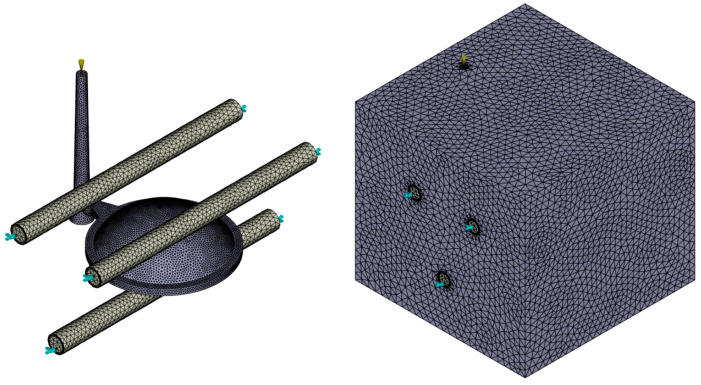
Meshed cooling system and injection mold block.

The next step in the simulation preparation involved creating the cooling system and the injection mold block (Figure 5). The cooling channel trajectories, including the inlets and outlets, were derived from the actual three-cavity mold design but were simplified for the simulation to cover a single cavity area. The core, cavity, and other mold plates were represented as an integrated block, sufficient for simulation purposes. These geometries were equipped with a suitably configured three-dimensional mesh, and its parameters were verified. Notably, the 3D mesh generated in Moldflow 2023 for each model was exported to Cadmould v17, enabling analyses beyond Moldflow’s capabilities.

When analyzing sink marks and thickness variations in thick-walled parts, results from Moldflow can be misleading. The software evaluates these phenomena based on the distribution of volumetric shrinkage and the configuration of the part’s geometry. It is designed to detect sink marks under ribs or in areas of local wall thickness increase. However, it is not intended for evaluating sink marks across large volumes as a whole. For purely thick-walled products, there will be excessive shrinkage, but the software solver will not detect sink marks and thickness changes due to the calculation method used. In contrast, the Cadmould v17 software can predict changes in thickness and sink mark depth even in areas with a large concentration of material volume for thick-walled parts. This capability allows for a more accurate assessment of potential defects in purely thick-walled regions, addressing the limitations present in other simulation tools.

After completing this step, it was necessary to set all necessary conditions required for the simulation, as listed in Table 2. Since the aim of the study was to vary the values of certain process parameters, their ranges are provided.

### 2.7. Procedure of 3D Scanning and Dimensional Inspection

Due to the difficulty of measuring the depth of sink marks, which represented a critical quality deficiency in the samples, conventional methods were insufficient. Therefore, to compare simulated deformations with the actual parameters of the injection-molded part, principles of three-dimensional scanning were utilized. This procedure involves scanning the surface of the sample using a laser 3D scanner, processing the resulting data, and comparing it with the geometry of the reference CAD model. When properly designed, this measurement and evaluation procedure allows for an efficient and reliable assessment of the parameter in question.

**Figure 6:**
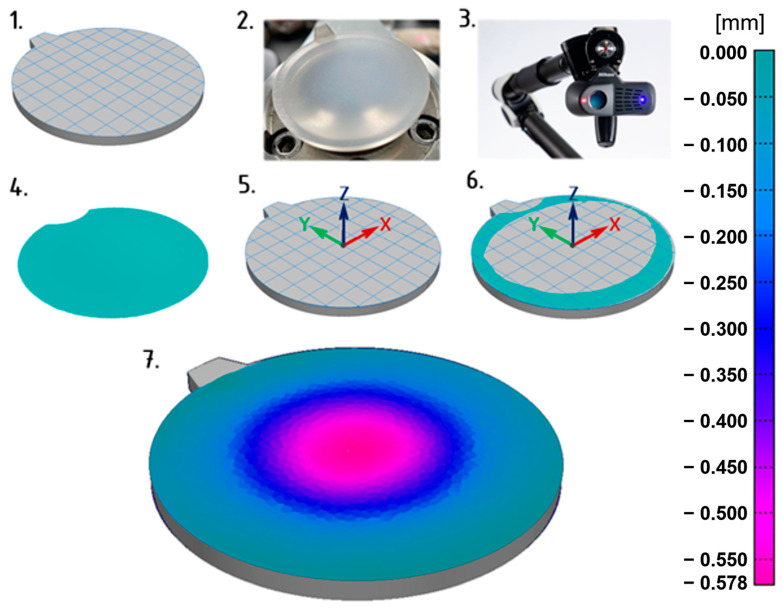
Procedure for scanning the surface of the test samples and evaluating sink mark depth.

Figure 6 illustrates the procedure that was applied. The initial step involved importing the reference CAD model into the Polyworks 2023 software environment. Given that the focus was on the depth of the sink mark on the underside of the lens, it was necessary to extract this planar surface from the 3D model (Step 1). Since the evaluation software is integrated in real-time with the scanner, this reference surface could be used as a limiting factor for the area to be scanned, meaning that regions outside this surface were ignored during scanning. This significantly saved time and effort related to processing the scanned data into the desired form.

Before scanning, the lens surface was coated with a very thin layer of anti-reflective spray (based on titanium dioxide) to prevent reflections that would hinder the acquisition of complete data of the scanned surface (Step 2). Without this step, it would be necessary to obtain the data through multiple scans or by correcting the scanned surface, complicating the process and reducing reliability. The scanning process then proceeded, with the scanning head mounted on the end of an arm and manually guided using laser guide beams to capture the entire surface from the required distance (Step 3). Once this step was completed, the scanned surface of the lens appeared in the software environment, ready for further processing (Step 4). Part of the scanned surface in the immediate vicinity of the gate was deliberately removed, as deformations caused by the separation of the gate residue could introduce unwanted errors and distort the surface.

The process of evaluating the sink mark depth from the reference surface extracted from the CAD model is illustrated in next steps. After defining the coordinate system (Step 5), which is configured such that the sink mark depth is measured in the negative *Z*-axis direction, the alignment of the scanned and reference surfaces was performed (Step 6). This alignment was achieved by fitting the undeformed perimeter of the scanned lens surface to the reference surface, establishing a zero level that served as the measurement baseline. All scanned geometry below this level is attributed to the sink mark depth, with the software rendering the deviation magnitudes using a color map, where the values can be read from the legend (Step 7). In this case, the maximum depth of the sink mark relative to the reference (neutral) surface is −0.578 mm. This method was applied consistently to measure the sink mark depths for all thicknesses of the injection-molded lenses.

### 2.8. Evaluation and Selection of the Gate Geometry

**Figure 7:**
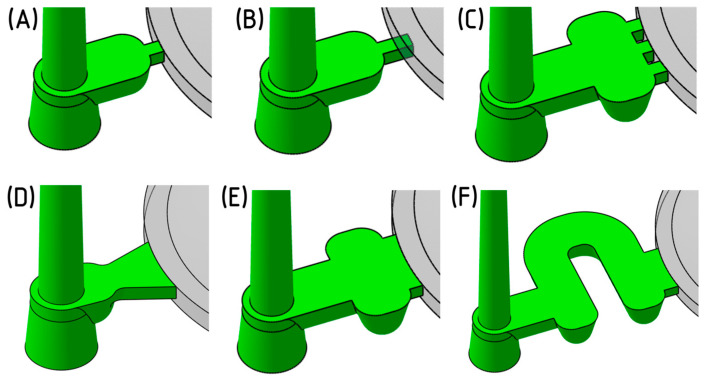
Designed modifications of gate geometries: (**A**): point gate; (**B**) sub-gate; (**C**) three-point gate; (**D**) fan gate; (**E**) film gate type 1; (**F**) film gate type 2.

The selection of the gate geometry for the experiment was preceded by Moldflow simulations, which were conducted and evaluated for six individual gate geometries, all considering a lens thickness of 16.5 mm. The gate geometries are categorized into point gates (Figure 7A–C) and film gates (Figure 7D–F). The primary requirements for the gate are to ensure uniform cavity filling with an appropriate flow type to avoid defects and to facilitate effective packing pressure. In terms of gate placement, optical requirements for the product must be considered, ensuring that the gate mark does not interfere with the functional surface of the part.

To compare the effectiveness of various gate types, three specific parameters were evaluated (Table 3). The process conditions and simulation settings were standardized across all cases to ensure that only the impact of gate geometry on the injection molding process was assessed.

**Figure 8:**
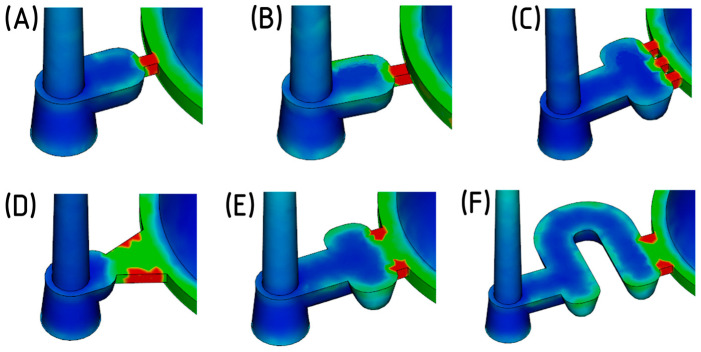
Thickness of the frozen layer as a fraction of the part thickness, captured 6 s after injection (red = solid; blue = melt): (**A**): point gate; (**B**) sub-gate; (**C**) three-point gate; (**D**) fan gate; (**E**) film gate type 1; (**F**) film gate type 2.

The gate solidification time (Figure 8) indicates the effective duration of the packing phase. A later solidification time allows the molten material to flow into the mold cavity for a longer period, thus better compensating for the shrinkage of the cooling material. Simulation results revealed that point gates fully solidified within 6 s from the start of injection. In contrast, fan or film gates extended the packing phase by almost 2 s.

Shear stress and shear rate are critical phenomena occurring during the flow of polymer melt. The maximum allowable values for these parameters are specific to each polymer and are typically outlined in the material data sheet. Exceeding these thresholds can cause excessive stress on the melt, leading to crack formation, undesirable internal stresses, and material degradation. The simulation results showed that the polymer melt experienced significantly lower shear stress when passing through the fan and film gates due to their larger cross-sections compared to the point gates.

**Figure 9:**
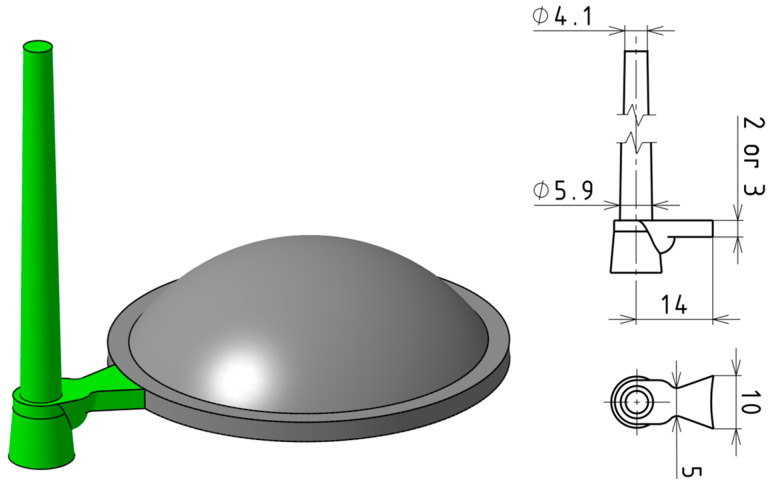
Designed gate system relative to the injection-molded test sample.

In light of these factors, the manufacturing and assembly aspects of each gate design were also assessed. The concept of a rotating gate insert holder, with the gate recessed in its front face, emerged as a desirable solution. By rotating the insert, the specific cavity to be filled can be easily defined. Although all proposed gate designs could potentially meet this requirement with certain modifications, the fan gate was ultimately selected. This gate type offers a sufficiently long packing phase duration, minimizes shear stress on the melt, is easy to manufacture, and allows for the simple adjustment of its thickness to either 2 or 3 mm (Figure 9).

## 3. Results

### 3.1. Initial Simulation Study

The aim of the initial simulation study was to determine the geometric defects and production times of the test samples based on their thickness, gate thickness, and the temperature characteristics of the melt and mold. This study provides an overview of how variations in these parameters affect the depth of sink marks, part deformation, and the time required to reach ejection temperature.

#### 3.1.1. Fill Time

Initially, the filling process of the mold cavity was evaluated, providing valuable information regarding the uniformity and flow rate of the polymer melt. In the injection molding of thick-walled optical lenses, it is crucial to ensure that the advancing melt front exhibits the desired fountain flow. According to the simulation results, this was achieved, preventing the occurrence of undesirable jetting, which can be particularly problematic in thick-walled parts.

**Figure 10:**
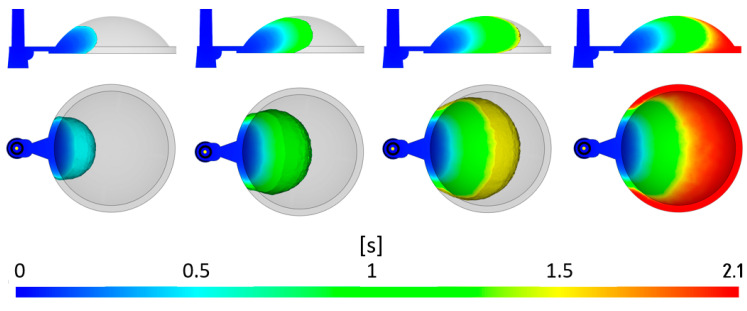
Visualization of mold cavity filling.

The sequences in Figure 10, captured at regular intervals during the cavity-filling process, also show that the flow is uniform, and the occurrence of defects caused by the collision of multiple melt fronts will be minimized. Additionally, it can be evaluated that the complete filling of the gate system and mold cavity occurs in approximately 2.1 s. Overall, it can be concluded that the designed concept of the gate system and mold cavity appears to be satisfactory, as do the selected injection material and initial process parameters.

#### 3.1.2. Sink Marks

**Figure 11:**
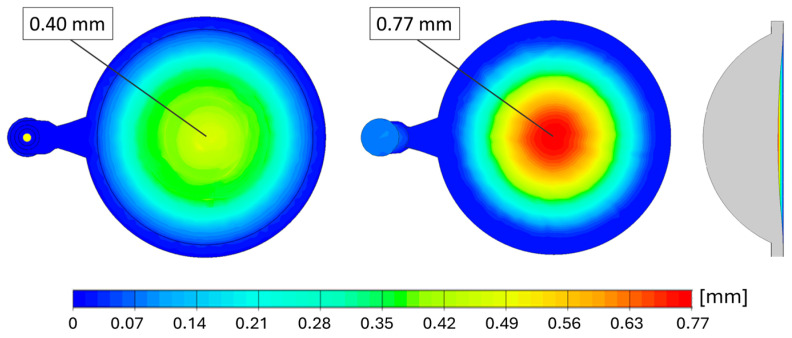
Simulation results indicating sink mark depth.

The results related to the occurrence and size of sink marks (Figure 11) were evaluated using Cadmould v17 software, which features a computational mode specifically designed for assessing the qualitative parameters of thick-walled parts. The obtained results largely correspond with those from the previous study on volumetric shrinkage. In the area with the highest material accumulation, where significant volumetric shrinkage is evident, a pronounced sink mark with a maximum depth of 0.77 mm is formed. This defect is observed on the back side of the lens. The shrinkage on the front side is significantly lower (0.40 mm) because less material is accumulated in this region, and the curvature of the lens surface limits the formation of sink marks.

This defect is therefore predominant on the planar back side of the lens, and is centrally located. In actual injection molding, this defect is likely to be even more pronounced, as the predicted result pertains to the state when the product is cooled to the ejection temperature of 130 °C. Further changes (additional shrinkage) will occur during subsequent cooling to ambient temperature (25 °C). Given the required function of the lens, the detected sink mark is unacceptable, and its depth must be minimized.

**Figure 12:**
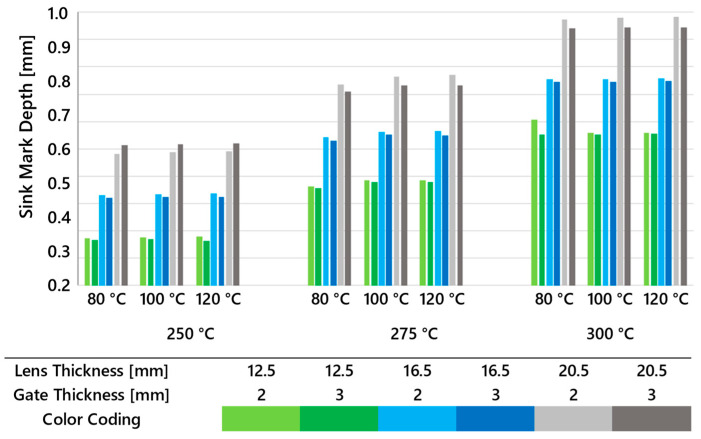
The depth of sink marks predicted by Cadmould calculated for all three lens thicknesses, two gate thicknesses, and multiple melt and mold temperature settings.

Figure 12 illustrates the predicted depth of the sink mark calculated by Cadmould v17 for three lens thicknesses, two gate thicknesses, and varying melt and mold temperatures. It is evident from the graph that the evaluated sink mark depth increases with lens thickness. When comparing the impact of melt temperature on sink mark depth, it becomes clear that higher temperatures result in deeper sink marks. This can be attributed to greater volumetric shrinkage and other phenomena that occur during cooling from the injection melt temperature to the ejection temperature. In terms of mold temperature, the evaluated data show no significant impact on the depth of the sink mark. Comparing results for different gate thicknesses reveals that a larger gate cross-section consistently results in a shallower sink mark. This is likely due to the more effective action of the packing phase compared to that in smaller cross-sections.

#### 3.1.3. Deflection

**Figure 13:**
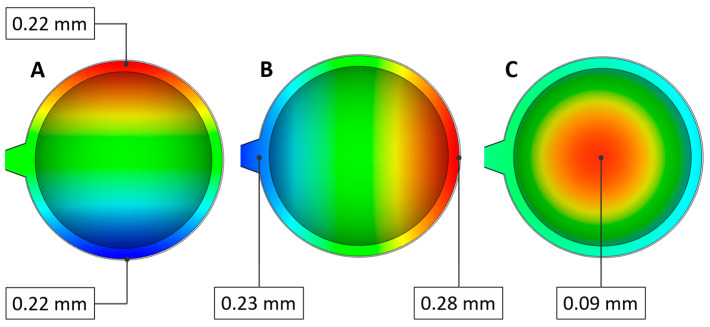
Evaluated maximal deformations ((**A**): X direction, (**B**): Y direction, (**C**): Z direction).

This Moldflow result represents the shape change of the injection-molded part compared to the reference CAD model geometry while maintaining a constant material volume (Figure 13). The influence of sink marks is not included. The magnitude of the reported deformations is calculated based on the different cooling rates and varying degrees of shrinkage. Deformations are visualized separately in the X, Y, and Z directions. The greatest deformation occurs in the Y direction on the side opposite the gate, while the smallest occurs in the Z direction at the center of the lens. In the X direction, symmetrical deviations are observed. Green-colored areas indicate nearly zero deformation compared to the reference model across all sub-results. This study shows that while deformations are not negligible, they contribute significantly less to reductions in lens quality compared to sink marks.

**Figure 14:**
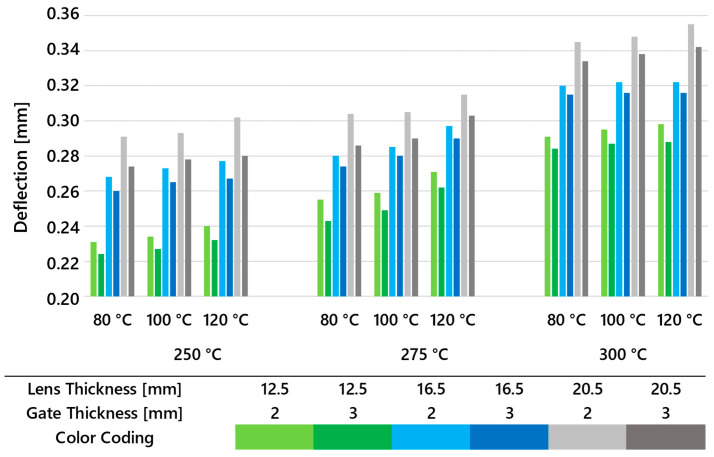
Deflection calculated for all three lens thicknesses, two gate thicknesses, and multiple melt and mold temperature settings.

Figure 14 illustrates the maximum deformations observed in all types of lenses when varying the mold and melt temperatures. It is evident that overall deformations increase with rising melt temperature, while increases in mold temperature contribute significantly less to dimensional deviations. However, when comparing gate thicknesses, it is clear that, in all cases, larger gate cross-sections result in smaller deformations, similar to the previously evaluated sink marks.

#### 3.1.4. Time to Reach Ejection Temperature

**Figure 15:**
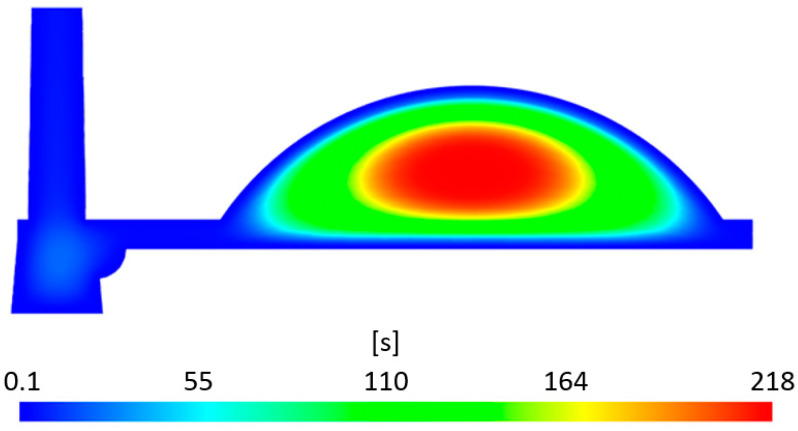
The time required to reach ejection temperature.

The Moldflow simulation result visualized in Figure 15 illustrates the time required to reach the ejection temperature, which is set at 130 °C for the material in question. It should be noted that the melt temperature in the simulation was set at 275 °C and the mold temperature at 100 °C. Given the thick-walled nature and uneven geometry of the sample, the results indicate significant heat retention in the central region of the lens where the thickness reaches 16.5 mm, leading to an increased time needed to achieve the target temperature.

In the central area, this value is predicted by the simulation to be 218 s from the start of the cycle, while the edge areas with a thickness of 3 mm reach the ejection temperature after just 10 s. This creates a considerable non-uniformity in the temperature profile across the lens thickness and results in different cooling rates. During cooling, the hot core in the form of melt will tend to pull in the surrounding, cooler material. Enhancing heat dissipation with an appropriate tempering system, using highly thermally conductive materials, and lowering the melt and mold temperatures could potentially reduce this time, although longer production cycles are to be expected for thick-walled parts.

**Figure 16:**
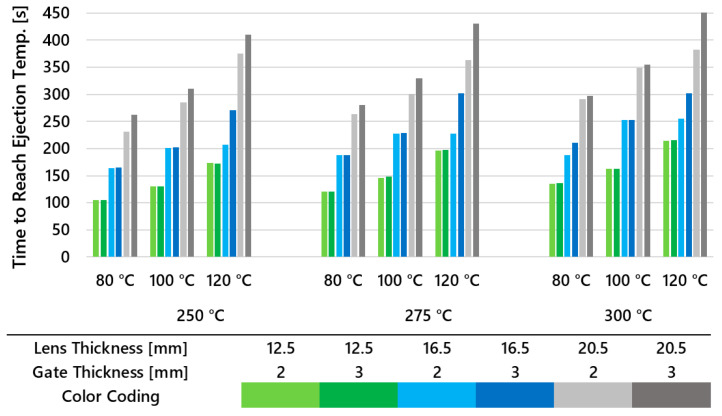
Time to reach ejection temperature calculated for all three lens thicknesses, two gate thicknesses, and multiple melt and mold temperature settings.

To gain an understanding of the time required to reach the ejection temperature for all three lens thicknesses using two gate system thicknesses and varying melt and mold temperatures, an extensive simulation was conducted incorporating these variables. The maximum times are plotted in Figure 16, allowing for the comparison of cooling times across the different variants. It is evident that with increasing lens thickness and mold temperature, the cooling time rises sharply. The influence of melt temperature and gate thickness, though less pronounced, is still significant, and becomes more evident in thicker lenses at higher melt temperatures.

#### 3.1.5. Summary of Initial Simulation Study

Overall, the simulation predictions suggest that injection molding should not result in inhomogeneities that cause significant visual defects. While the size of deformations under constant volume is not negligible, this contributes far less to the overall dimensional instability of the product compared to the volumetric changes causing sink marks on the back side of the lens. Therefore, the primary objective is to reduce the depth of these sink marks to the lowest possible minimum. It is anticipated that this will also minimize deformations, as both phenomena are closely related. Since the simulation results indicate that a gate thickness of 2 mm leads to a higher degree of undesirable effects, further experiments focused primarily on injection molding using a 3 mm gate thickness.

### 3.2. Reduction in Apparent Defects during Test Sample Preparation

**Figure 17:**
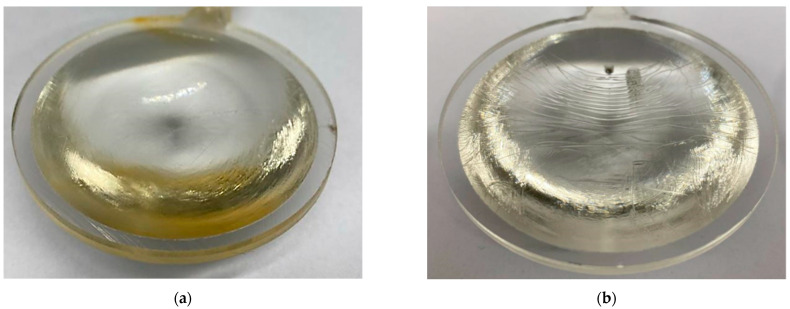
Images of test samples from the initial test series: (**a**) severe material yellowing; (**b**) noticeable flow marks on the surface.

During the initial injection molding series aimed at verifying the functionality of the mold, several types of defects were observed in addition to sink marks and deformations. The most common defects included yellowing, melt flow marks, and air entrapment leading to bubble formation (Figure 17). The initial injections were conducted at a melt temperature of 275 °C and a mold temperature of 100 °C. Injection pressure, packing pressure, and injection speed values were varied to identify a range of acceptable minimum and maximum values to be applied as boundary conditions for the experimental design.

**Figure 18:**
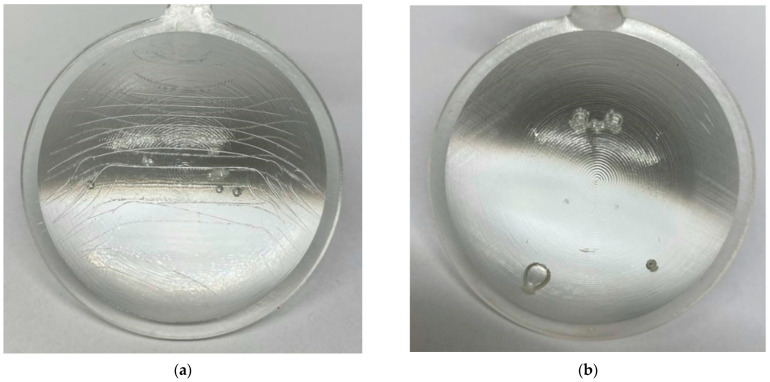
Images of test samples after partial correction of process parameters: (**a**) persistent flow lines; (**b**) air bubbles.

Injection pressures exceeding 100 MPa and packing pressures above 80 MPa resulted in significant shear stress on the melt as it passed through the gate, leading to thermal degradation which resulted in the yellowing of the material. Conversely, injection pressures below 60 MPa caused visible flow marks in the direction of melt flow on both the front and back sides of the lens. Another notable defect was the presence of air bubbles within the lens volume (Figure 18), which occurred when the injection speed was set higher than 20 mm/s. These threshold values were verified for all three lens thickness variants and across the ranges of mold and melt temperatures specified by the material manufacturer.

### 3.3. Specification of Tested Process Parameter Ranges

The selection of tested process parameter ranges in this study was based on both material data sheet recommendations and the initial experimental molding trials. We aimed to ensure that the apparent defects mentioned earlier would not occur during test sample preparation, allowing only dimensional deviations to be measured and evaluated.

During the initial molding trials, various levels and ranges of injection and packing pressure were tested to establish an acceptable range that allowed for further variations in melt and mold temperatures, as well as sample thicknesses, while avoiding apparent defects. To maintain consistency in the experiment, the level of packing pressure was set during the preparation of all test samples to 48 or 80 MPa, which is 5% above the recommended processing conditions defined by the material’s manufacturer. Based on previous simulation results, it was evident that a higher and prolonged effect of the packing phase leads to a reduction in volumetric changes during the cooling of the material, thus reducing sink mark depth. Accordingly, the aim was to determine the highest acceptable injection pressure.

However, excessively high packing pressure can induce residual stresses within the part, leading to issues such as warping, cracking, and distortion over time. Significant cracks from the surface to the center of the lens were clearly visible in some samples prepared with injection pressures over 100 MPa. Overpacking and high residual stress can negatively impact the optical properties and overall dimensional stability of the part. High pressure also causes increased shear heating and material degradation, resulting in changes in material properties such as reduced clarity and increased yellowing (almost browning) of the material, as well as increased brittleness. These effects were notably observed during sample preparation with pressures exceeding 100 MPa. High packing pressure can result in birefringence, where the material exhibits different refractive indices in different directions, degrading the optical performance of the part, especially in applications requiring precise light transmission or focusing. Although this could potentially happen, in this study, optical properties were not examined, and further research on residual stress and birefringence effects is needed.

Conversely, lower injection and packing pressures resulted in voids and air traps, compromising the part’s structural integrity and optical properties, which were observed with pressures lower than 60 MPa. Additionally, the surface finish was compromised, leading to a dull or uneven appearance that negatively affected the optical clarity and aesthetic quality. This issue was also partially observed during the trials. Most critically, the excessive formation of sink marks was prevalent, highlighting the need for higher pressure settings.

Regarding the selection of melt and mold temperatures, the absolute minimum and maximum values were derived from the material manufacturer’s data sheet and recommended processing conditions. During the initial molding trials, broader temperature ranges exceeding these recommendations were also tested to explore their effects. Melt temperatures higher than 300 °C immediately resulted in the excessive thermal degradation of the material, even at the lowest pressures and injection rates. Conversely, temperatures lower than 250 °C led to several issues. Firstly, the material solidified too quickly during flow, creating visible flow lines and weak weld lines, compromising both the appearance and structural integrity of the part. Incomplete filling occurred as the material did not flow properly into all areas of the mold, leading to short shots and inadequate plasticization. This premature solidification also reduced the duration of the packing phase due to early gate solidification.

For mold temperature, exceeding the recommended minimum of 80 °C led to several problems. Low mold temperatures caused the surface of the part to solidify too quickly, resulting in a rough or uneven surface finish that reduced optical clarity and aesthetic quality. Rapid cooling caused differential shrinkage, increasing residual stresses within the part, which could lead to warping, cracking, or dimensional instability over time. Additionally, the premature solidification of the gate reduced the effectiveness of the packing phase. On the other hand, mold temperatures above the maximum recommended value of 120 °C resulted in surface defects such as gloss variations, surface haze, and even burning and thermal degradation marks, which negatively impacted optical clarity and aesthetic quality. Prolonged exposure to high temperatures caused the thermal degradation of the polymer, leading to discoloration, reduced mechanical properties, and compromised optical quality. High mold temperatures also affected the cooling rate and molecular orientation of the polymer, leading to birefringence, where the material exhibited different refractive indices in different directions, compromising optical performance.

**Figure 19:**
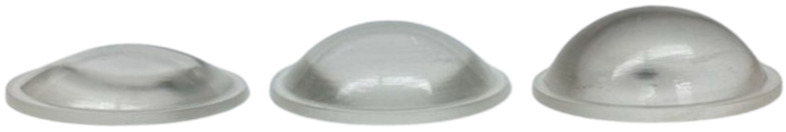
Manufactured test samples (lenses) with thicknesses of 12.5 mm, 16.5 mm, and 20.5 mm without visible defects.

Thus, the selected ranges of melt and mold temperatures were based on balancing the need to avoid these defects while ensuring desirable processing conditions. The aim was to find a range that allowed sufficient variation to study their effects comprehensively while avoiding apparent defects and ensuring the highest possible product quality. By adhering to the specified ranges of process parameters listed below, all three lens thicknesses could be produced without exhibiting the aforementioned defects (Figure 19). These minimum and maximum values can thus be considered for experimental design, with variations aimed at minimizing the depth of the resulting sink marks, which will be measured using reverse engineering principles. The verified ranges of the process parameters were as follows: injection pressure 60–100 MPa, packing pressure 48–80 MPa, injection speed 20 mm/s, melt temperature 250–300 °C, and mold temperature 80–120 °C.

### 3.4. Design of Experiment (DOE)

**Figure 20:**
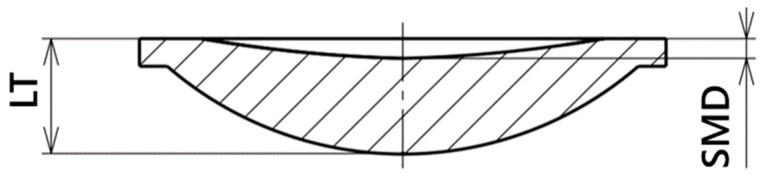
Evaluation of sink mark depth (SMD) in relation to lens thickness (LT).

Using Minitab 21 software, experimental conditions were designed and a subsequent data evaluation was conducted, incorporating both simulation results and the values obtained from the laser scanning of injection-molded lenses. The objective of the experiments was to vary the injection molding process conditions to achieve the lowest possible depth of the observed quality parameter, which was the sink mark depth on the back side of the lens (Figure 20). The simulation results were effectively utilized to identify statistically significant process parameters and their combinations, with settings aimed at minimizing the sink mark depth. The identified optimal conditions were then verified through the actual production of test samples and the measurement of the actual sink mark depth. This approach allowed for the determination of the process conditions that minimize the impact of sink marks and established the degree of deviation between the software simulation predictions and the actual injection-molded samples.

To determine the appropriate experimental conditions, the concept of applied statistics DOE (Design of Experiment) was selected to achieve optimal input parameter values. A DOE design with three factors and two levels was chosen for the study (Table 4). The number of experimental repetitions was set to one, as this is a simulation experiment, and the number of blocks was also limited to one to maintain consistent experimental conditions. The input factors investigated included pressure, melt temperature, and mold temperature, with the output variable being the sink mark depth obtained from the simulation. The injection process is initially controlled by a flow rate of 19.5 cm^3^/s until 99% of the cavity is filled. At this point, the control switches to pressure control, where the quoted injection pressure of 60 or 100 MPa is applied. This means that the defined injection pressure has a significant effect primarily during the packing phase, where the pressure level is set to 80% of the maximum injection pressure, which is 48 or 80 MPa. Each input factor was tested at two levels (minimum and maximum values). This approach was applied separately for the three considered lens thicknesses. By systematically varying these parameters, the goal was to minimize the depth of the sink marks and ensure the dimensional stability of the thick-walled polycarbonate optical lenses.

Table 5 presents the design of the input parameters along with the predicted sink mark depths. Based on the specified variations, eight simulations (runs) were conducted, with process parameters set according to the values provided. The resulting data were then used to evaluate the examined sink mark depths. This enabled further analysis and assessment of the significance of the effects of individual factors and their combinations.

#### 3.4.1. Determining the Significance of Individual Tested Factors

**Figure 21:**
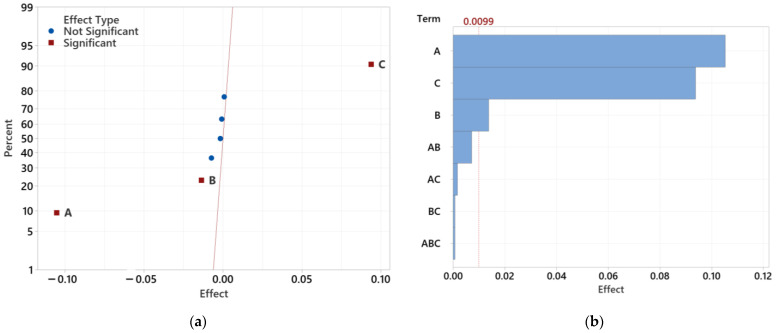

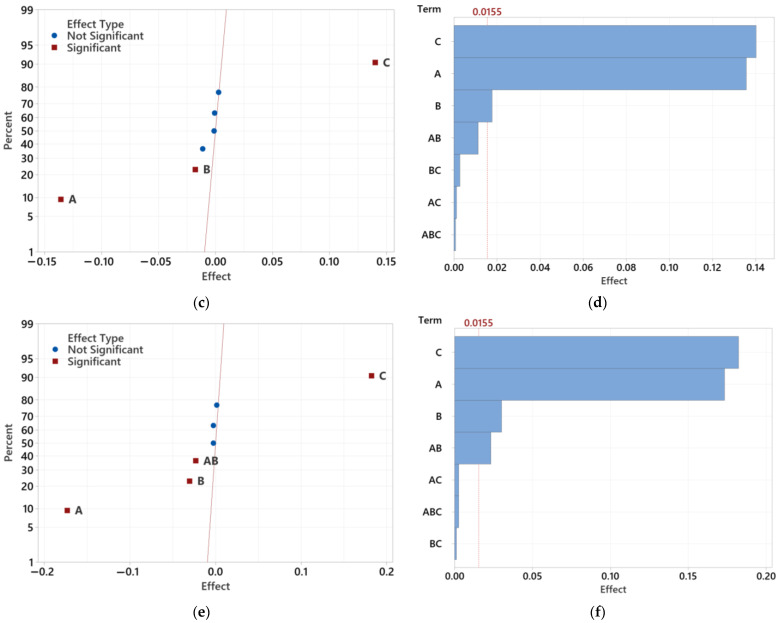
The effects of all factors and their combinations including Pareto charts: (**a**) effect of factors for 12.5 mm lens; (**b**) Pareto chart for 12.5 mm lens; (**c**) effect of factors for 16.5 mm lens; (**d**) Pareto chart for 16.5 mm lens; (**e**) effect of factors for 20.5 mm lens; (**f**) Pareto chart for 20.5 mm lens.

Figure 21 illustrates the magnitude, direction, and significance of the effects of individual factors and their interactions for all thicknesses of the lenses. Statistical significance is determined by the distance from the zero axis, with significant and insignificant effects differentiated by the color and shape of symbols. The graph shows that injection pressure, mold temperature, and melt temperature are statistically significant factors. Injection pressure and mold temperature have a negative correlation with the observed value, meaning that increasing these parameters decreases the observed value. In contrast, melt temperature has a positive effect, increasing the observed value.

Due to the division of the graph into positive and negative influences, comparing the actual impact of statistically significant parameters may be challenging. Therefore, a Pareto chart was created for each test sample to compare the relative magnitude and statistical significance of the main and interaction effects, with the reference line (shown in red) indicating statistically significant effects.

For the 12.5 mm thick lens, injection pressure, mold temperature, and melt temperature are significant at the α = 0.05 level, with injection pressure being the most significant factor. For the 16.5 mm thick lens, injection pressure and mold temperature negatively correlate with the observed values, while melt temperature positively affects them. In this case, melt temperature emerges as the most significant factor. This can be attributed to the fact that, with larger sample thicknesses, volumetric changes occurring during the cooling of the melt are more dominant than the effects of pressure and packing.

In the 20.5 mm thick sample, melt temperature and injection pressure are the most significant factors, while mold temperature and interactions between factors are less significant. Notably, the combination of injection pressure and mold temperature (AB) reached statistical significance after the initial evaluation without further reduction.

Overall, the Pareto charts indicate that melt temperature has the highest statistical significance, followed by injection pressure and mold temperature. The combination of pressure and mold temperature also reaches statistical significance and is most significant among all studied cases, with other combinations being less relevant for minimizing sink mark depth.

**Figure 22:**
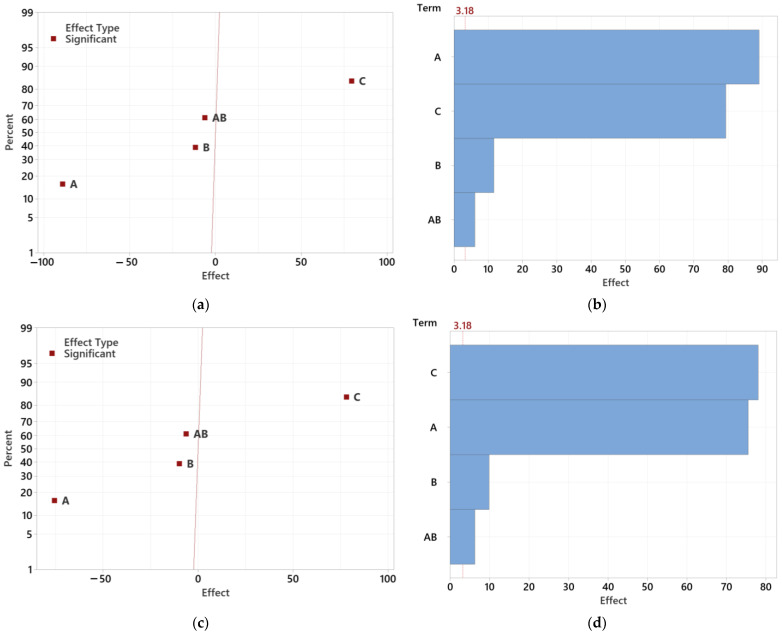

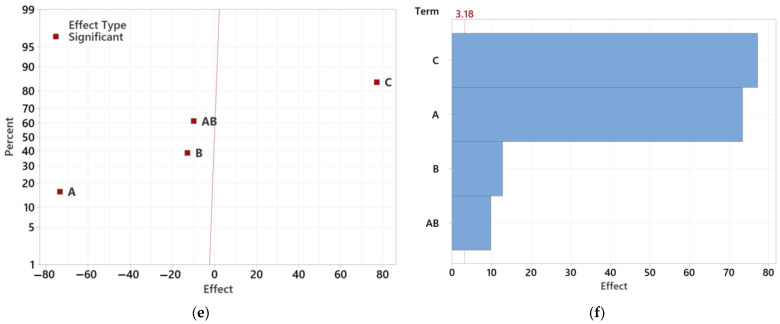
The effects after reducing insignificant factors and their combinations, including Pareto charts: (**a**) effect of factors for 12.5 mm lens; (**b**) Pareto chart for 12.5 mm lens; (**c**) effect of factors for 16.5 mm lens; (**d**) Pareto chart for 16.5 mm lens; (**e**) effect of factors for 20.5 mm lens; (**f**) Pareto chart for 20.5 mm lens.

The next step involved reducing statistically insignificant factors (Figure 22). Following the initial analysis of effect influences for the 12.5 mm sample, statistically insignificant factors were gradually removed, and the analysis was repeated until only factors significant at the α = 0.05 level remained. These results show that after eliminating insignificant effects, the combination of pressure and mold temperature (AB) became statistically significant. Although this combination has a limited effect on sink mark depth, it is statistically significant to consider.

To better visualize the significance of individual effects, a Pareto chart of standardized effects was also created. The level of statistical significance was influenced by the removal of insignificant effects. For the 12.5 mm samples, injection pressure remains the most significant factor, while the combination of pressure and mold temperature is the least significant.

Similarly, the analysis for the 16.5 mm sample involved reducing statistically insignificant factors. After the initial evaluation, insignificant effects were eliminated, retaining only statistically significant factors. The combination of pressure and mold temperature (AB) became significant, although it has a limited impact on sink mark depth.

The process was repeated to clearly identify the most influential process parameters. The obtained results align closely with the previous findings, demonstrating that melt temperature, injection pressure, mold temperature, and their interactions with injection pressure are statistically significant.

**Figure 23:**
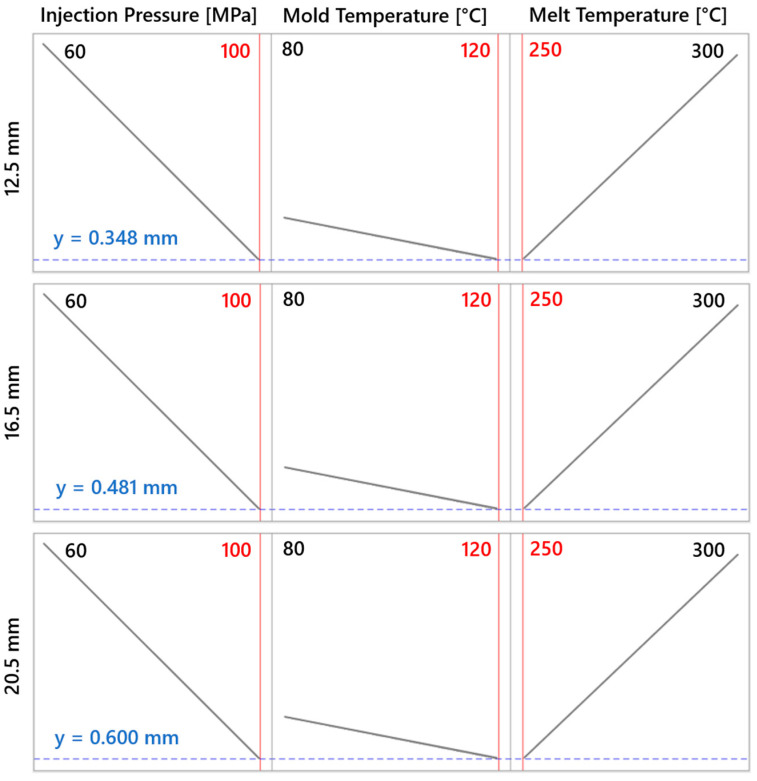
Response optimizer results for all test sample thicknesses.

Figure 23 visualizes the response optimizer used to find the lowest sink mark depth for samples of different thicknesses. For the 12.5 mm thick sample, an injection pressure of 100 MPa, mold temperature of 120 °C, and melt temperature of 250 °C were identified as optimal, resulting in a minimum-achievable sink mark depth of y = 0.347 mm. Similarly, the optimizer calculated the minimum-achievable sink mark depth for other samples.

#### 3.4.2. Overview of DOE Findings

In accordance with the Design of Experiment (DOE) methodology, experiments were designed and conducted at the simulation level for all sample thicknesses. The results obtained were subsequently used to optimize the process parameters, with the primary aim of minimizing sink mark depth. In all cases, it was found that increasing injection pressure and mold temperature leads to a reduction in sink mark depth. Conversely, decreasing the melt temperature also reduces the extent of sink marks.

As seen in Table 6, for the 12.5 mm thick lens, injection pressure is the dominant factor, while for the other thicknesses, melt temperature has the most significant impact. The significance of other factors is similar across all samples. Generally, increasing the melt temperature enhances the thermal expansion of the material. When the polymer melt is injected into the mold and rapidly cooled, it shrinks. Greater thermal expansion at higher temperatures results in more significant volumetric shrinkage. The pronounced effect of melt temperature on sink mark depth for thicker samples can be attributed to the longer cooling time to the ejection temperature, leading to more substantial volumetric changes, slightly outweighing the influence of injection pressure and the subsequent packing phase. However, these aspects are most significant for the 12.5 mm thick lens, where the compensation of volumetric changes during packing shows the highest statistical significance. The mold temperature, in all cases, has a lower impact on sink mark formation but remains above the statistical significance threshold. Higher mold temperatures can lead to less thermal contraction of the material during cooling, potentially reducing volumetric shrinkage. In contrast, lower mold temperatures slow the cooling process, potentially causing greater volumetric shrinkage and more significant sink marks.

The minimum-achievable sink mark depths, given the optimal process parameter settings, are derived from the injection process simulations (Table 7). Based on the current DOE study using two levels for each process parameter, it is suggested that lower sink mark depths were not observed within these specific ranges. However, this does not conclusively demonstrate that lower values could not be achieved at temperatures or pressures between the tested levels, as the study did not explore intermediate values. The next section of the research focuses on validating these predictions through the actual injection molding of samples under optimal process parameters and the subsequent measurement of actual sink mark depth. This will allow the determination of the minimum sink mark depths achievable with the considered input parameters.

### 3.5. Statistical Evaluation of the Experiment

Based on the DOE methodology, simulations were designed and conducted for all sample thicknesses. The results enabled the optimization of process parameters to minimize sink mark depth. For each lens thickness, the simulations identified the lowest achievable minimum and the specific process conditions at which this minimum was obtained. These values then served as the conditions for producing test samples on which the actual sink mark depth was measured. The obtained data were further evaluated and compared with the simulation values, allowing for the determination of the discrepancy between simulation and reality.

The subsequent evaluation was conducted consistently across all three optical lens thicknesses in terms of applied statistical methods, the production of samples, and the measurement of sink mark depth. This ensured consistency in assessing the influence of process parameters on the resulting sink mark depth, although the evaluation was performed separately for each thickness.

During the filling phase, the process was controlled by a flow rate of 19.5 cm^3^, with a switch to pressure control at 99% cavity filling. At this point, the quoted injection pressure of 60 or 100 MPa was applied. The effect of injection pressure is therefore significant primarily during the packing phase, where it is 80% of the injection pressure (48 or 80 MPa).

The injection of the samples was carried out under the process parameter settings that yielded the lowest possible sink mark depths according to the optimizer. For all sample thicknesses, the conditions were an injection pressure of 100 MPa, a mold temperature of 120 °C, and a melt temperature of 250 °C. Under these conditions, a total of 30 samples were produced for each thickness, providing a sufficient sample size for the adequate statistical analysis of the actual sink mark depths.

#### 3.5.1. Analysis of Statistical Data for 12.5 mm Thick Lens

**Figure 24:**
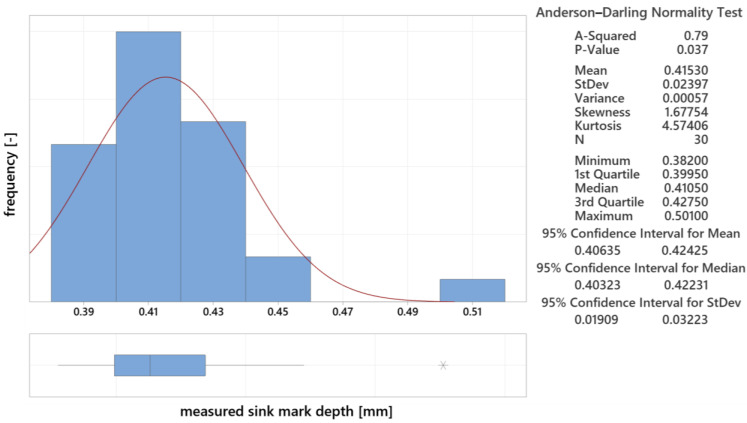
Measured data summary for 12.5 mm thick lens.

The initial evaluation of the measured parameters used the Graphical Summary function in Minitab 21 (data are visualized in Figure 24). This included the mean (0.415 mm), standard deviation (0.024 mm), and coefficient of variation. The bar graph with a Gaussian curve shows the frequency of measurements, while the box plot identifies outliers. An outlier was detected, and it was excluded from the following analysis. The adjusted mean was 0.412 mm, with a standard deviation of 0.018 mm.

**Figure 25:**
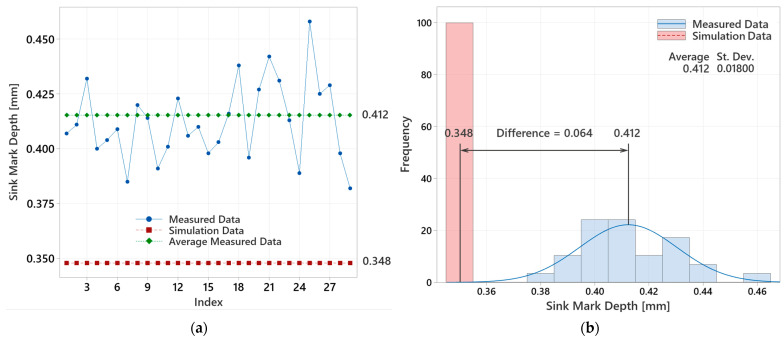
Evaluation of measured and simulated data for 12.5 mm thick lens: (**a**) measured values versus simulated results; (**b**) analysis of average measured value and simulation outcome.

For the overall evaluation of the experiment with a 12.5 mm lens, it was necessary to determine the operational optimum and compare the actual measured data (sink mark depth) with the values obtained from the injection molding simulations. Figure 25 illustrates the measured values (blue dots), their average (green squares), and the simulation-predicted values (red squares). These results reveal the differences between the mean values of the actual measurements and the simulated predictions.

These differences indicate that the actual measured data and the simulation results are not aligned, suggesting that the discrepancies are statistically significant. This variance could be attributed to the ideal and unchanging conditions in the simulations. The discrepancy may be due to the limitations in the simulation software’s ability to accurately predict the sink mark depth, especially for thicker lenses. These limitations could stem from the software’s assumptions and simplifications that do not fully capture the real-world complexities of the injection molding process. The exact reasons for the increasing discrepancy with thicker lenses cannot be confirmed at the moment, as detailed access to the simulation computing methods and mathematical models used in the software is not available. Therefore, further investigation into the simulation algorithms and their handling of post-ejection cooling would be necessary to fully understand these differences.

#### 3.5.2. Analysis of Statistical Data for 16.5 mm Thick Lens

**Figure 26:**
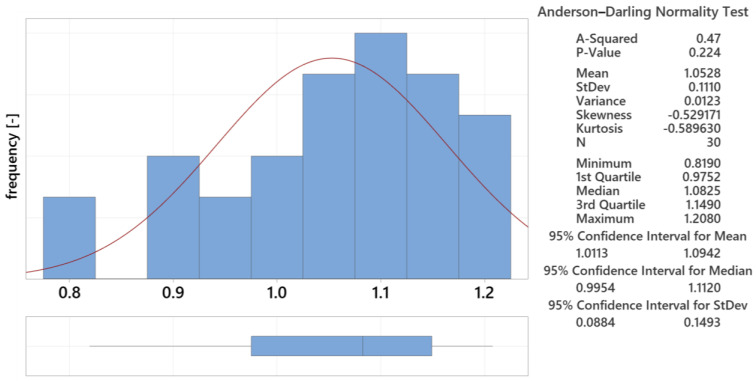
Measured data summary for 16.5 mm thick lens.

Using the Graphical Summary function, the 16.5 mm lens data were summarized, including the mean (1.053 mm), standard deviation (0.111 mm), and coefficient of variation, as visualized in Figure 26. Grubbs’ test revealed no outliers, confirming the data’s alignment with the expected distribution and ensuring reliability.

**Figure 27:**
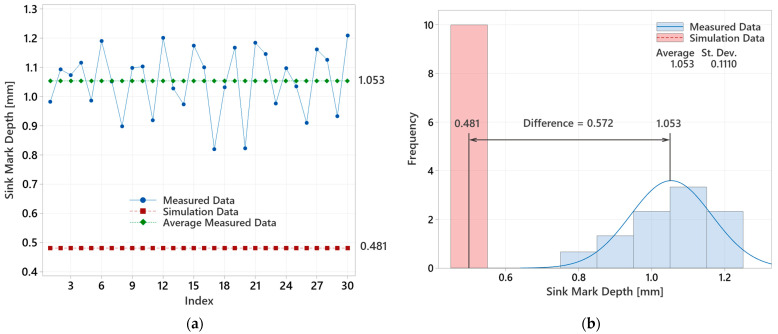
Evaluation of measured and simulated data for 16.5 mm thick lens: (**a**) measured values versus simulated results; (**b**) analysis of average measured value and simulation outcome.

Similarly, to comprehensively evaluate the experiment with a 16.5 mm lens, the operational optimum was identified, and the actual measured sink mark depths were compared with the values derived from the injection molding simulations. Figure 27 highlights the significant discrepancy between the mean measured values and the simulation results.

The marked differences suggest that the discrepancies are not random. This could be due to the idealized conditions in the simulations and the point at which the simulated sink mark depth is assessed, which does not account for the additional shrinkage during cooling to ambient temperature. Moreover, the discrepancy may be more evident for thicker lenses. Additionally, the inaccuracies in the material data within the software database could also contribute to the observed deviations. The hypothesis regarding material inaccuracy is based on discussions with software specialists, who suggested that discrepancies in material data could sometimes reach up to 10%. Specifically, inaccuracies in thermal properties such as thermal conductivity and specific heat, as well as mechanical properties like modulus of elasticity and shrinkage rates, could significantly impact the simulation outcomes. In all simulations, the predicted sink mark depths were consistently lower than the measured values. This consistent underestimation suggests that the material properties used in the simulations may not fully capture the behavior of the actual material during the injection molding process. However, this hypothesis has not been experimentally validated. Future studies should consider varying these material properties within reasonable ranges to determine if this can bring the simulation results closer to the experimental data.

#### 3.5.3. Analysis of Statistical Data for 20.5 mm Thick Lens

**Figure 28:**
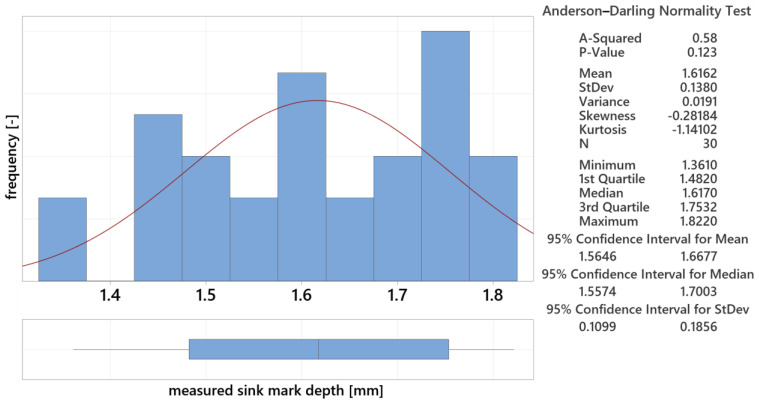
Measured data summary for 20.5 mm thick lens.

For the 20.5 mm lens, the Graphical Summary provided statistical data, including the mean (1.616 mm), standard deviation (0.138 mm), and coefficient of variation, as shown in Figure 28. The bar graph and Gaussian curve illustrate the data distribution, and Grubbs’ test found no outliers, confirming data validity.

**Figure 29:**
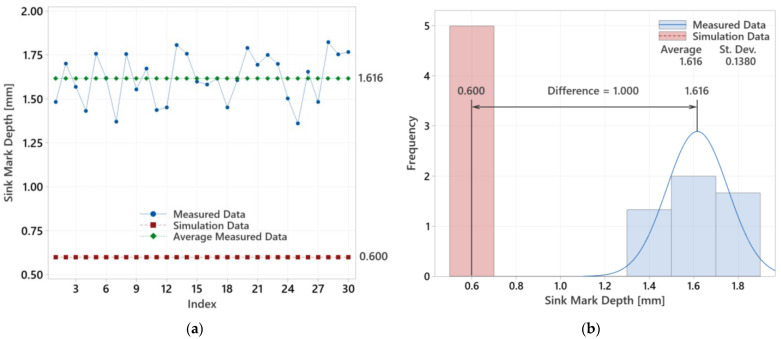
Evaluation of measured and simulated data for 20.5 mm thick lens: (**a**) measured values versus simulated results; (**b**) analysis of average measured value and simulation outcome.

For the final evaluation with a 20.5 mm lens, the operational optimum was determined, and the actual measured sink mark depths were compared with the simulated values. Figure 29 displays the measured values, their averages, and the simulation-predicted values. The graphs show discrepancies between the actual measured values and the simulation results.

#### 3.5.4. Summary of Statistical Evaluation

Based on the Design of Experiment (DOE) method, simulations were conducted for various sample thicknesses to optimize process parameters and minimize sink mark depth in lens injection molding. These simulations identified the minimum-achievable sink mark depth for each thickness and the specific process conditions required. The data from these simulations guided the production of test samples, on which actual sink mark depths were measured and compared to simulation results.

The Graphical Summary function provided visualizations and basic statistical calculations, including mean, standard deviation, and coefficient of variation. Grubbs’ test checked for outliers. A statistical analysis confirmed significant differences between the actual measurements and the simulations, likely due to idealized simulation conditions and the neglect of additional shrinkage post-ejection. Further experimental validation is necessary to confirm the optimal process parameters derived from the simulations and to understand the physical aspects not captured by the simulation models.

**Figure 30:**
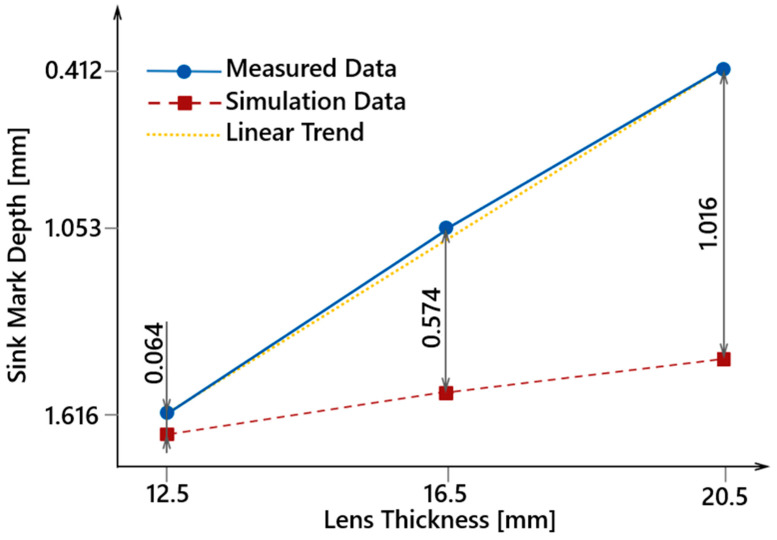
Comparison of measured data with ideal linear trend and simulation data.

Table 8 presents the minimum-achievable sink mark depths for all lens thicknesses as determined by the simulation optimization, along with the average measured actual sink mark depths for comparison. It is evident that both the simulated and actual sink mark depths increase with sample thickness, with the deviation between actual and simulated values also increasing linearly. For the 12.5 mm lens, the difference is 0.064 mm; for the 16.5 mm lens, it is 0.574 mm; and for the 20.5 mm lens, it is 1.000 mm. Confirming this linear trend (Figure 30) would require additional measurements across more lens thicknesses, but for the purposes of this study, the conducted measurements were sufficient.

## 4. Discussion

This study focused on an experimental analysis of the injection molding process for thick-walled optical polycarbonate lenses, emphasizing the influence of process parameters on product quality. Initial software analysis investigated mold cavity filling, packing, cooling, and dimensional instabilities. Simulations in Moldflow 2023 and Simcon Cadmould v17 provided detailed insights into these processes, identifying potential defects and key factors affecting product quality.

The results showed that the simulations could predict and identify potential defects, such as homogeneous cavity filling, without significant visual flaws. Monitoring the effective packing phase and mold thermal field offered additional insights into the solidification and shrinkage processes. Evaluating deformations, volumetric shrinkage, and sink mark depth enabled a comprehensive assessment of dimensional aspects. While deformations at a constant volume were notable, they were less impactful compared to the volumetric changes causing sink marks.

Our primary objective was to minimize sink mark depth, assuming this would concurrently reduce deformations. Discovering that a 2 mm gate caused undesirable effects led to further research using a 3 mm gate. This finding, along with the simulation results, was crucial for optimizing the injection process to produce high-quality optical lenses with minimal defects.

Practical experiments confirmed that adhering to specified process parameters eliminated significant defects like yellowing, flow marks, and bubbles. These experiments provided insights into the conditions for producing thick-walled optical lenses with reduced dimensional and shape deviations, based on the process parameters defined by the simulation outcomes. The series of experiments aimed to minimize sink mark depth, with the simulations providing statistically significant information on influential process parameters.

The analysis revealed that injection pressure and mold temperature significantly influence sink mark formation, with injection pressure being more dominant for 12.5 mm lenses and melt temperature for thicker samples. Lower melt temperatures reduced sink mark depth due to decreased thermal contraction during cooling. The results indicated that simulations might be prone to inaccuracies, particularly for thicker samples. Paired T-tests confirmed significant differences between real measurements and simulations, with discrepancies increasing with sample thickness.

The relationship between sink marks and warpage in injection-molded thick-walled polycarbonate optical lenses is complex and influenced by several interrelated factors. Both phenomena are primarily driven by the volumetric shrinkage that occurs during the cooling phase of the molding process. Sink marks are surface depressions that form when the outer surface solidifies while the inner material continues to shrink, pulling the surface inward. Warpage, on the other hand, is the deformation or twisting of the part caused by differential shrinkage rates throughout the part, leading to internal stresses and geometric instability.

Our experimental results indicate that both sink marks and warpage are significantly affected by the same process parameters: injection pressure, packing pressure, melt temperature, and mold temperature. Specifically, higher packing pressures and longer packing times reduce the formation of sink marks by compensating for the material’s volumetric shrinkage. However, these same conditions can induce residual stresses that contribute to warpage if not carefully controlled.

For instance, higher injection and packing pressures (up to 100 MPa) effectively minimized sink marks by ensuring better material flow and compensation for shrinkage. However, excessive pressures led to increased residual stresses, resulting in the cracking of the samples. This is consistent with the theoretical understanding that higher pressures enhance material packing but can also increase the potential for differential shrinkage, leading to warpage and other defects.

Lower melt temperatures (250 °C) reduced sink marks by decreasing the thermal expansion and contraction during cooling. Conversely, lower mold temperatures (down to 80 °C) led to rapid cooling and increased residual stresses. Optimizing these temperatures is crucial; while higher mold temperatures (up to 120 °C) reduced residual stresses and warpage, they also increased the risk of surface defects and thermal degradation. Generally, increasing the melt temperature enhances the thermal expansion of the material. When the polymer melt is injected into the mold and rapidly cooled, it shrinks. Greater thermal expansion at higher temperatures results in more significant volumetric shrinkage. The pronounced effect of melt temperature on sink mark depth for thicker samples can be attributed to the longer cooling time (up to 452 s) to the ejection temperature, leading to more substantial volumetric changes, slightly outweighing the influence of injection pressure and the subsequent packing phase. However, these aspects are most significant for the 12.5 mm thick lens, where the compensation of volumetric changes during packing shows the highest statistical significance.

The mold temperature, in all cases, has a lower impact on sink mark formation but remains above the statistical significance threshold. Higher mold temperatures can lead to less thermal contraction of the material during cooling, potentially reducing volumetric shrinkage. In contrast, lower mold temperatures would accelerate the cooling process, potentially causing greater volumetric shrinkage and more significant sink marks.

In conclusion, the combined experimental and simulation approach offers a comprehensive understanding of the injection molding process for optical lenses. Optimizing process parameters based on simulations provides valuable guidelines for manufacturing with minimal defects. However, monitoring and comparing results with real experimental data is crucial to validate simulation accuracy and establish a reliable production process. The relationship between sink marks and warpage is governed by the interplay of volumetric shrinkage and residual stresses. Future research should aim to quantify these interactions in greater detail and explore advanced process control techniques to achieve even more favorable outcomes.

## 5. Conclusions

In conclusion, this study offers significant insights into the injection molding of thick-walled polycarbonate optical lenses. The research focused on varying lens thicknesses and utilized simulations with Autodesk Moldflow 2023 and Simcon Cadmould v17, demonstrating that film gate designs are superior to point gates for uniform cavity filling and effective packing. While the simulations provide a robust theoretical framework, experimental validation highlighted discrepancies between the predicted and actual sink mark depths, especially for thicker lenses. These findings suggest that further experimental studies are needed to refine the simulation models and fully validate the optimal process parameters. Experimental validation corroborated the reliability of these simulations, providing crucial data on actual material behavior during molding. These findings facilitate the optimization of process parameters and gate designs to achieve high-quality lenses with minimal defects. Key parameters such as melt temperature, mold surface temperature, injection pressure, and packing pressure were found to significantly influence the dimensional and shape stability of the lenses, including their optical properties. This study not only enhances theoretical understanding, but also offers practical guidance for industrial applications in the manufacturing of thick-walled optical components.

## Figures and Tables

**Table 1:** Three-dimensional mesh parameters of molded part.

Parameters	Thickness of Test Sample		
	12.5 mm	16.5 mm	20.5 mm
Number of Elements	220,265	324,226	489,055
Connected Nodes	33,924	59,362	86,800
Maximum Element Aspect Ratio	11.37	14.86	16.14
Minimum Element Aspect Ratio	1.04	1.05	1.03
Average Element Aspect Ratio	2.64	2.83	3.28
Volume of Tetra Elements	19.51 cm^3^	24.39 cm^3^	29.96 cm^3^
Maximum Dihedral Angle	172.7°	173.1°	173.5°

**Table 2:** Defined simulation parameters.

Selected Simulation Parameters	
Injection Molded Material	PC Makrolon LED 2245 (Covestro AG, Leverkusen, Germany)
Mold Material	Tool Steel P20 (1.2311) (Meusburger Georg GmbH + Co KG, Wolfurt, Austria)
Coolant	Oil (8 L/min)
Coolant Channel Diameter	8 mm
Injection Molding Machine	Arburg Allrounder 470 E 1000-290 (Arburg GmbH + Co KG, Loßburg, Germany)
Melt Temperature Values	250 °C, 275 °C, 300 °C
Mold Temperature Values	80 °C, 100 °C, 120 °C
Ejection Temperature	130 °C
Filling Time	2 s
Injection Pressure	Automatically
Velocity/Pressure Switchover	at 99% of Cavity Volume Filled
Packing Pressure	80% of injection pressure (48 or 80 MPa)
Packing Time	10 s

**Table 3:** Summary of simulation results for determining the suitability of gate geometry.

Gate Type	Gate Freeze Time [s]	Shear Stress [MPa]	Shear Rate [1/s]
Point (A)	5.9	0.93	9760
Sub-gate (B)	5.7	1.02	11,760
Three-point (C)	5.6	0.66	3690
Fan gate (D)	7.6	0.46	3780
Film type 1 (E)	7.7	0.49	3130
Film type 2 (F)	7.5	0.39	2670

**Table 4:** Considered input factors and their levels.

Input Factor	Process Parameter	Test Level 1 (Min. Value)	Test Level 2 (Max. Value)	Unit
A	Injection Pressure	60	100	MPa
B	Mold Temperature	80	120	°C
C	Melt Temperature	250	300	°C

**Table 5:** DOE for all sample thicknesses including sink mark depth responses from simulations.

Run Order	Injection Pressure [MPa]	Mold Temperature [MPa]	Melt Temperature [MPa]	Sink M. Depth Lens 12.5 [mm]	Sink M. Depth Lens 16.5 [mm]	Sink M. Depth Lens 20.5 [mm]
1	60	80	250	0.466	0.636	0.802
2	100	80	250	0.369	0.512	0.652
3	60	120	250	0.458	0.626	0.791
4	100	120	250	0.348	0.481	0.600
5	60	80	300	0.560	0.774	0.983
6	100	80	300	0.461	0.649	0.833
7	60	120	300	0.555	0.771	0.980
8	100	120	300	0.440	0.622	0.778

**Table 6:** Comparison of statistical significance of evaluated factors.

Order of Statistical Significance	Lens 12.5 mm	Lens 16.5 mm	Lens 20.5 mm
1.	A	C	C
2.	C	A	A
3.	B	B	B
4.	AB	AB	AB

**Table 7:** Summary of the results from the optimizer tool.

Evaluated Parameter	Lens 12.5 mm	Lens 16.5 mm	Lens 20.5 mm
Min. Sink Mark Depth [mm]	0.348	0.481	0.600
Injection Pressure [MPa]	100	100	100
Mold Temperature [°C]	120	120	120
Melt Temperature [°C]	250	250	250

**Table 8:** Comparison of measured sink mark depths on test samples with simulated results.

Lens Thickness [mm]	Measured Sink Depth [mm]	Simulated Sink Depth [mm]	Difference [mm]
12.5	0.412 ± 0.018	0.348	0.064
16.5	1.053 ± 0.111	0.481	0.572
20.5	1.616 ± 0.138	0.600	1.061

## Data Availability

The original contributions presented in the study are included in the article, further inquiries can be directed to the corresponding author/s.
